# Other PHAC publications

**DOI:** 10.24095/hpcdp.45.1.05

**Published:** 2025-01

**Researchers from the Public Health Agency of Canada also contribute to work published in other journals and books. Look for the following articles published in 2024:**

Archambault PM, Rosychuk RJ, Audet M, Hau JP, Graves L, Dcary S, […] **Aziz S, Zakaria D**, et al. Post-COVID-19 condition symptoms among emergency department patients tested for SARS-CoV-2 infection. Nat Commun. 2024;15(1):8449. https://doi.org/10.1038/s41467-024-52404-4

**Chen JC**. Lessons from a data scientist during COVID-19. Nat Microbiol. 2024;9(10):2466-7. https://doi.org/10.1038/s41564-024-01815-6

Christopher G, Biswas A, **Lang JJ, Prince SA**. Occupational and sex differences in active commuting among Canadian workers from 2006 to 2016. Health Rep. 2024;35(9):1-15. https://doi.org/10.25318/82-003-x202400900001-eng

Devane D, Hamel C, Gartlehner G, Nussbaumer-Streit B, Griebler U, Affengruber L, […] **Garritty C**. Key concepts in rapid reviews: an overview. J Clin Epidemiol. 2024;175:111518. https://doi.org/10.1016/j.jclinepi.2024.111518

**Doyon-Plourde P, Farley R, Krishnan R, Tunis M**, Wallace M, **Zafack J**. Direct quantitative comparison of benefits and risks of COVID-19 vaccines used in National Immunization Technical Advisory Groups Guidance during the first two years of the pandemic. Vaccine. 2024;42(26):126406. https://doi.org/10.1016/j.vaccine.2024.126406

Drolet M, Laprise J, Chamberland , Sauvageau C, Wilson S, Lim GH, […] **Ashleigh Tuite**, et al. Switching from a 2-dose to a 1-dose program of gender-neutral routine vaccination against human papillomavirus in Canada: a mathematical modelling analysis. CMAJ. 2024;196(33):E1136-43. https://doi.org/10.1503/cmaj.240787

**Hajo S, Capaldi CA, Liu L**. Sexual and gender minority youth in Canada: An investigation of disparities in positive mental health. Can J Public Health. 2024. https://doi.org/10.17269/s41997-024-00931-4

**Killikelly A, Siu W, Abrams EM, Salvadori MI**. Respiratory syncytial virus vaccination in older adults. CMAJ. 2024;196(29):E1011. https://doi.org/10.1503/cmaj.240906

Mirza AIi, Zhu F, **Knox N**, Black LJ, Daly A, **Bonner C, Van Domselaar G**, et al. Mediterranean diet and associations with the gut microbiota and pediatric-onset multiple sclerosis using trivariate analysis. Commun Med (Lond). 2024;4(1):148. https://doi.org/10.1038/s43856-024-00565-0

Turcotte L, **Scott MM**, Petrcich W, Tanuseputro P, Kobewka D. Quality of advance care planning in long-term care and transfers to hospital at the end of life. J Am Med Dir Assoc. 2024;25(11):105259. https://doi.org/10.1016/j.jamda.2024.105259

Vasiliadis HM, Roberge P, Spagnolo J, Lamoureux-Lamarche C, Chapdelaine A, Brodeur M, […] **Ishimo MC**, et al. A digital iCBT intervention for social anxiety disorder in Quebec and Ontario: protocol for a multi-phase effectiveness-implementation study. BMC Psychiatry. 2024;24(1):662. https://doi.org/10.1186/s12888-024-06082-7

**Yao X, Rama AA, Mazzitelli J, McFaull SR, Thompson W**. A mixed methods study on poisoning and injury-related emergency department visits associated with opioids in Canada, 2011 to 2022: from the Canadian hospitals injury reporting and prevention program. BMC Public Health. 2024;24(1):2546. https://doi.org/10.1186/s12889-024-20016-8

